# A Comparison of the Incidence and Type of Ocular Motility Defects in Patients Presenting to Birmingham Midland Eye Centre Emergency Department during 2019 and 2020 to Assess the Impact of the Covid-19 Pandemic

**DOI:** 10.22599/bioj.258

**Published:** 2022-09-20

**Authors:** Jessica Edwards, Eilidh Russo, Rosie Auld

**Affiliations:** 1 Birmingham and Midland Eye Centre, GB

**Keywords:** COVID-19, Orthoptics, Pandemic, Emergency Department, NHS

## Abstract

**Aim:**

This audit aimed to investigate whether COVID-19 had any impact on the incidence and type of ocular motility defects in patients presenting to Birmingham Midland Eye Centre (BMEC) Emergency Department (ED) during the COVID-19 pandemic in 2020 compared to the previous year.

**Methods:**

Medical records were reviewed for all patients presenting to BMEC ED during 2019 and 2020. Patients were classified depending on their diagnosis. The incidence and classification of ocular motility defect were analysed. Factors considered during analysis were number of presentations by year and month; COVID-19 tests; and pre-existing conditions.

**Results:**

Two hundred and twenty-one patients presented in 2019, and 260 patients in 2020, an increase in incidence of 17.6% was observed. One hundred and eighty-five patients were classified with new-onset neurogenic conditions in 2019, and 222 patients in 2020, an increase of 20.0%. In 2020, most patients presented in July, November, and December. Overall, there was a 91.3% increase in new-onset fourth cranial nerve palsies in 2020. Fifty-seven patients in 2020 had a Polymerase Chain Reaction COVID-19 test, of these 5 were COVID-19 positive.

**Conclusion:**

There was a higher incidence of ocular motility defects in 2020 compared to 2019. The majority of ocular motility defects were classified as neurogenic. It is difficult to attribute this increase to COVID-19 due to lack of testing and results, and confounding variables such as pre-existing conditions and lockdown restrictions. Some potential explanations for the change in presentations across the year of 2020 are proposed.

## Introduction

An outbreak of a severe acute respiratory syndrome coronavirus 2 (SARS-CoV-2) started in Wuhan City, Hubei Province of China. Common symptoms of Coronavirus-19 (COVID-19) include fever, cough, fatigue, and shortness of breath (Guan et al. 2020). Severe infections are known to cause pneumonia and can even be fatal. More severe infections have been reported in patients with pre-existing medical conditions (Huang et al. 2020).

Neurological manifestations occurring alongside COVID-19 infection have been reported. Mao et al. (2020) reported that central nervous system (CNS) and peripheral nervous system (PNS) manifestations occurred after COVID-19 infection. CNS symptoms of dizziness and headaches were more prevalent than PNS symptoms of taste and smell impairment. Acute disseminated encephalomyelitis (ADEM), a rare demyelinating disease, due to the COVID-19 virus was also reported (Parsons et al. 2020).

Neurogenic conditions with COVID-19 presenting as ocular motility defects have been reported in the literature including case studies of third, fourth, and sixth nerve palsies (Dinkin et al. 2020, Belghmaidi et al. 2020, Ordás et al. 2020, Francis 2021). Internuclear ophthalmoplegia (INO) with oculomotor nerve palsy (Gutiérrez-Ortiz et al. 2020) and oscillopsia in association with Bickerstaff encephalitis (Llorente Ayuso et al. 2021) have also been reported after COVID-19.

The pathophysiology of the neurogenic manifestations from COVID-19 is currently not well understood. The entry point of the virus into the cell has been reported as the receptor angiotensin-converting enzyme 2 (ACE2) on the cell surface (Groß et al. 2020). An overexpression of ACE2 in the lungs explains why respiratory problems are most common with COVID-19 infection (Groß et al. 2020). Neurological damage linked with COVID-19 infection may be explained by ACE2 receptors being observed in nerve cells (Mao et al. 2020).

COVID-19 had a significant impact on hospital services during 2020. Birmingham Midland Eye Centre (BMEC), a tertiary ophthalmic centre, remained open for urgent patients who presented to the Emergency Department (ED). This allowed for a comparison in the incidence of patients who attended in 2019—a regular year, and in 2020—the first year during the COVID-19 pandemic. This audit aimed to investigate whether COVID-19 had any impact on the incidence of all patients who presented to the Orthoptic Department from BMEC ED with ocular motility defects.

## Method

Case notes were reviewed for all new patients presenting to BMEC ED where an orthoptic assessment was completed between 1st January 2019 to 31^st^ December 2020. An orthoptic report was requested by a triage nurse when a patient had binocular diplopia or a new eye movement disorder. Patients were excluded if they were deemed orthoptically satisfactory (no ocular motility defect or binocular single vision disorder), had orbital trauma, or had symptoms for longer than six months. A retrospective analysis of these patients was performed.

Information was collected from the electronic patient records (EPR) and compiled in a Microsoft Excel spreadsheet. Due to the EPR, no notes were missing. For each case the following was recorded: orthoptic diagnosis, pre-existing conditions, and history of known or suspected COVID-19 testing and results. Pre-existing conditions were identified, documented, and analysed for each patient. This included cardiovascular diseases, high cholesterol, diabetes, and hypertension if known at time of presentation. Ocular motility defects were classified using the diagnostic criteria shown in Table 1.

**Table 1:** Classification of diagnoses of ocular motility defects.


CLASSIFICATION	DIAGNOSIS

**Concomitant**	Concomitant strabismus, convergence insufficiency, convergence spasm.

**Decompensated**	Decompensated heterophoria, decompensated longstanding fourth nerve palsy.

**Mechanical**	Graves’ orbitopathy, mechanical restrictions of extraocular muscles, acquired Brown’s syndrome.

**Myogenic**	Ocular Myasthenia.

**Acute Neurogenic**	New-onset third cranial nerve palsy, fourth cranial nerve palsy, sixth cranial nerve palsy, isolated muscle palsy, INO, supranuclear palsy, or multiple neurogenic condition.


The incidence of all patients presenting to BMEC ED was analysed for all classifications of ocular motility defect in 2019 and 2020.

## Results

Following exclusions, a total of 481 patients presented to BMEC ED with ocular motility defects in 2019 and 2020. There were 187 (38.9%) women and 294 (61.1%) men. The mean age of patients was 61.0 years (range 17–93 years, SD ± 17.5 years).

### Presentations to ED

**Figure 1:**
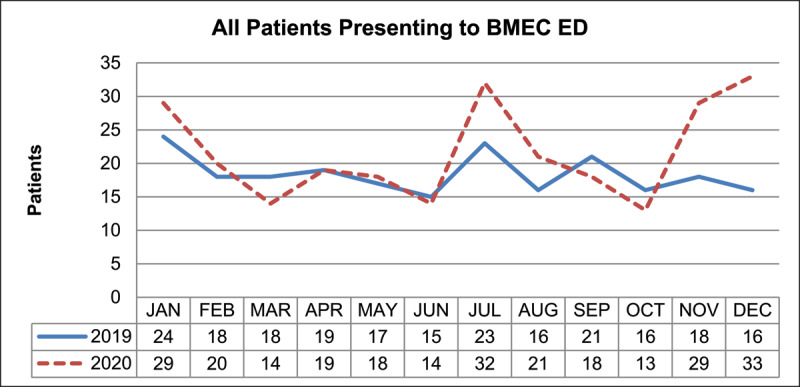
The number of patients presenting to BMEC ED with ocular motility defects by month in 2019 and 2020.

A total of 221 patients presented in 2019, and 260 patients presented in 2020, which was a percentage increase of 17.6%. More patients presented in July (39.1% increase), November (61.1% increase), and December (106.3% increase) in 2020 compared to 2019 (Figure 1).

The breakdown of the classifications of different ocular motility defects are shown in Table 2. One hundred and eighty-five patients presented with new-onset neurogenic conditions in 2019, and 222 patients in 2020, an increase of 20.0%. There was also a 50% increase in the number of patients presenting with decompensated strabismus in 2020.

**Table 2:** The Classification of patients attending BMEC Orthoptic Department.


	NEUROGENIC	CONCOMITANT	DECOMPENSATED	MECHANICAL	MYOGENIC

**2019**	185	5	12	13	6

**2020**	222	6	18	9	5


**Figure 2:**
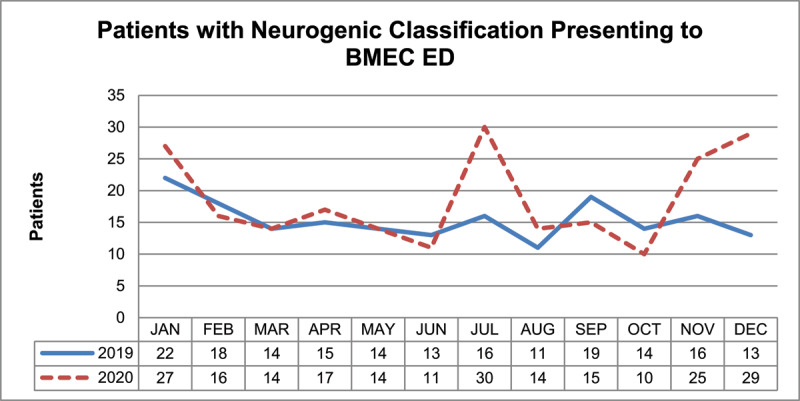
The number of patients presenting to BMEC ED with neurogenic ocular motility defects by month in 2019 and 2020.

The majority of patients presenting to BMEC ED who were seen in the Orthoptic Department, in both years, had neurogenic ocular motility defects. Four hundred and seven patients out of 481 (84.6%) were classified as neurogenic. The highest number of neurogenic presentations in 2020 occurred during the months of July, November, and December 2020 (Figure 2), with percentage increases of 87.5%, 56.3%, and 123.1%, respectively, when compared to the same months in 2019.

Overall, there were more new-onset fourth cranial nerve palsies, sixth cranial nerve palsies, isolated muscle palsies, and multiple cranial nerve palsies in 2020 compared with 2019, with the largest increase (91.3%) in fourth cranial nerve palsies (Table 3).

**Table 3:** Incidence of new-onset neurogenic ocular motility defects by year.


NEUROGENIC CLASSIFICATION	2019	2020	PERCENTAGE CHANGE (%)

**Third Cranial Nerve Palsy**	38	34	–10.5

**Fourth Cranial Nerve Palsy**	23	44	91.3

**Sixth Cranial Nerve Palsy**	90	109	21.1

**Isolated Muscle Palsy**	5	6	20.0

**INO**	11	5	–54.5

**Supranuclear Palsy**	4	2	–50.0

**Multiple Cranial Nerve Palsies**	14	22	57.1


### Pre-Existing Conditions

There was a similar percentage of patients with pre-existing conditions in 2020 compared with 2019 (Table 4).

**Table 4:** Percentage of patients with at least one pre-existing condition.


PERCENTAGE OF PATIENTS WITH AT LEAST ONE PRE-EXISTING CONDITION			

	2019	2020

All patients	66.1	64.6

Neurogenic patients	68.6	69.8


**Figure 3:**
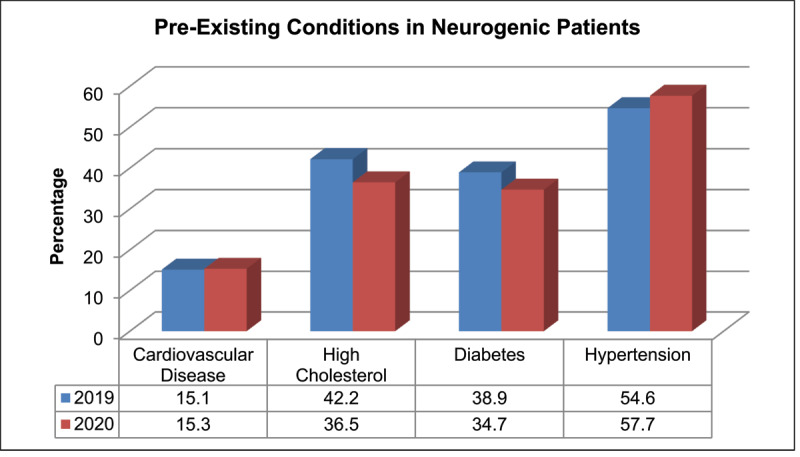
Percentage of patients with pre-existing conditions with neurogenic classification.

The percentage of patients in 2019 and 2020 with new-onset neurogenic ocular motility defects presenting with a risk factor are shown in Figure 3. Risk factors included a history of cardiovascular disease, high cholesterol, diabetes, and hypertension. The most common pre-existing risk factor was hypertension.

### COVID-19 testing

Diagnostic tests to identify COVID-19 were introduced in 2020, including the Polymerase Chain Reaction (PCR) and the Lateral Flow Test (LFT). When a PCR test was completed within the BMEC ED, results could be accessed from the EPR. Of the 260 patients who attended in 2020, 57 patients (21.9%) had a PCR COVID-19 test performed. Of these patients, 5 (8.7%) had positive result for COVID-19 (Table 5).

**Table 5:** COVID-19 Test Results in 2020.


COVID-19 TEST	TOTAL	NEGATIVE	POSITIVE

**No**	203	N/A	N/A

**Yes**	57	52	5


### Timeline of events in 2020

**Figure 4:**
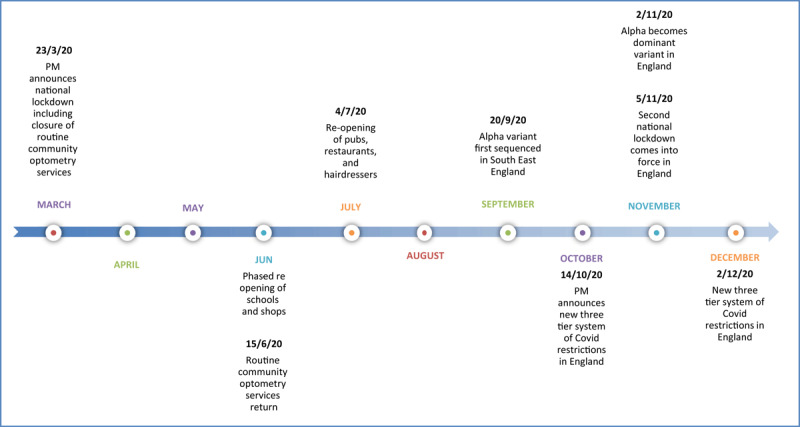
Timeline of events in 2020 (Institute for Government Analysis 2021, Nagra et al. 2021, NHS England, 2020b, Gov.uk 2021a, Public Health England 2021).

A summary timeline of events relating to COVID-19 in England is shown in Figure 4.

## Discussion

This audit of patients attending BMEC ED during 2020 aimed to investigate whether COVID-19 had an impact on the incidence of ocular motility defects. The patients seen in Orthoptic Department in 2020 with ocular motility defects were compared to the previous year, pre-pandemic (2019). The results of this audit should be considered in the context of the events in England during 2020, as summarised in Figure 4.

### Incidence

The incidence of COVID-19 in England, and the UK, varied throughout the year of 2020. The first wave of COVID-19 was at its peak in April 2020 (Kirby 2021), and then the second wave caused primarily by the Alpha variant saw increasing cases in November 2020 up to January 2021 (Gov.uk 2021a). The Prime Minister announced the national lockdown on the 23^rd^ March 2020 (Gov.uk 2020), which consequently caused a 25% reduction to all A&E visits the subsequent week (Thornton 2020). Prior to the gradual easing of restrictions from June 2020 (Institute for Government Analysis 2021), the emergency services in England saw an overall reduction in attendances, particularly in April (NHS England 2020a). This audit did not follow the national trend of attendances to ED in 2020 as outlined above, but instead found spikes of attendances in the months of July, November, and December, which implies they cannot be explained by the timings of the initial national lockdown.

Most patients attending BMEC ED during 2019 and 2020 were classified with neurogenic ocular motility defects, which consisted of all infranuclear, internuclear, nuclear, and supranuclear defects. The number of patients with neurogenic classification increased by 20.0% in 2020 compared to 2019, the highest incidences occurred in July, November, and December 2020, with the last two months being consistent with the rise in positive COVID-19 cases in the UK caused by the Alpha variant (Gov.uk 2021a).

Another consideration when auditing the presentations to ED was the access to primary optometry services during 2020. On 23^rd^ March 2020, optometry services were advised to stay open to deliver emergency eye care only (Nagra et al. 2021), and re-open for routine sight tests on 15^th^ June 2020 (NHS England, 2020b). The ED at BMEC saw an increase in presentations in July 2020, which coincided with the reopening of community optometry services. This may explain the rise in cases in July, as this was a month where the level of COVID-19 infection in the UK population was relatively low (Gov.uk 2021a), but more patients would have been able to attend community optometry services. It is possible that more patients attending reopened community optometry services led to increased numbers attending ED, following referral or advice from optometrists, for example suffering from binocular diplopia.

### Types of Defect

The incidence of infranuclear ocular motility defects increased in 2020 compared to 2019. This was most profound for new-onset fourth cranial nerve palsies, with a 91.3% increase in incidence in 2020. The reason for this increase in fourth cranial palsies is not yet known. There are relatively few studies describing fourth cranial nerve palsies alongside COVID-19 infection. Ordás et al. (2020) reported a case of trochlear nerve palsy with tonic pupil shortly after COVID-19 infection, but were unable to prove this was not due to a pre-existing microvascular condition. Doblan et al. (2021) evaluated all cranial nerve involvement in COVID-19 and found 1.7% of patients had trochlear nerve palsy after positive COVID-19 infection.

In this audit, an increase of 21.1% was found for the incidence of sixth cranial nerve palsies in 2020 compared to 2019. Francis (2021) reported a case of isolated abducens nerve palsy with anosmia in a 69-year-old woman with no known general health conditions after COVID-19 infection. This would suggest that COVID-19 may affect the function of the cranial nerves and may explain the increased incidence in fourth and sixth cranial nerve palsies at BMEC orthoptic department in 2020.

This audit also found a 57.1% increase in patients with multiple neurogenic ocular motility defects in 2020. Two studies have reported patients with multiple neurogenic disorders and COVID-19. Dinkin et al. (2020) reported bilateral abducens nerve palsy with left oculomotor nerve palsy and Gutiérrez-Ortiz et al. (2020) reported right INO with right oculomotor nerve palsy both from COVID-19 infection, which led to Miller Fisher syndrome.

The incidence of mechanical and myogenic conditions decreased in the 2020 cohort of this audit. Current literature suggests that COVID-19 could act as a trigger to autoimmune conditions such as myasthenia gravis and Graves’ disease (Mateu-Salat et al. 2020; Restivo et al. 2020). Restivo et al. (2020) reported three cases of positive COVID-19 with raised Acetylcholine receptor (AChR) antibodies, where all patients developed ocular symptoms such as ptosis and diplopia. However, this audit found a decrease in incidence of mechanical or myogenic conditions suggesting COVID-19 has not influenced the overall numbers of patients presenting with these conditions.

A small increase in the number of patients presenting to BMEC ED with decompensated heterophorias was found in 2020 compared to 2019. There are many factors that can contribute to decompensation of a heterophoria, including uncorrected refractive error, vision changes, age, and general health. Stevens (2021) reported a case study of a decompensated esotropia following COVID-19 infection that improved three weeks after onset, suggesting the infection may have caused the decompensation to occur. However, some studies report extended computer use can lead to diplopia due to the decompensation of a heterophoria (Maddii 2018). This audit cannot comment on the reason for the increase due to limited COVID-19 information, but the increase may be attributed to the influence of the COVID-19 pandemic, such as the increase in working from home and increase in general screen time with the lack of opportunities to go outside.

### Pre-Existing Conditions

A large proportion of the patients who presented to BMEC ED with neurogenic ocular motility defects had pre-existing conditions; 68.6% in 2019, and 69.8% in 2020. The most common pre-existing condition in all patients was hypertension. Guo et al. 2020 suggested patients with microvascular conditions were more likely to get severe COVID-19 infections and become hospitalised. Deaths from COVID-19 have also been associated with suffering from co-morbidities such as diabetes, hypertension, lung disease, and cardiovascular disease (Guo et al. 2020). Chen et al. 2021 evaluated the effect COVID-19 had on hospitalised patients without pre-existing hypertension. Elevated systolic blood pressure was observed in most of the patients, where diastolic was normal, and 8.42% of patients had a rise in blood pressure during hospitalisation. As poor control of microvascular conditions is a known cause for new-onset neurogenic ocular motility defects (Tamhankar et al. 2013), and COVID-19 has been shown to increase hypertension, this may be a factor related to the increased incidence of neurogenic ocular motility defects seen in 2020, but the lack of COVID-19 infection data in all cases makes it difficult to draw firm conclusions or associations.

### COVID-19

The pathophysiological mechanisms of the COVID-19 virus are currently not well understood. Studies suggest that some neurological manifestations could occur because of an aberrant immune response to COVID-19 (Gupta & Weaver 2021). Doblan et al. (2021) looked at the effect of COVID-19 on the cranial nerves but were unable to say whether the virus directly affected the nerves as some cranial nerves were affected and others in close proximity were not. There were a higher number of patients with loss of taste or smell, but patients were less likely to have ophthalmoparesis, hearing, or vision loss overall.

In this audit there were 57 patients (21.9%) who had a COVID-19 test in 2020, and of these, only 5 patients tested positive. There was limited LFT and PCR testing available in England, particularly in the first wave, where only inpatients and essential workers were eligible for COVID-19 tests up until the end of May 2020. After May 2020, anyone in England with symptoms was able to have a COVID-19 test (Gov.uk 2021b); therefore, there may have been unidentified COVID-19 positive patients during the first wave. As the pandemic continued, more people were having COVID-19 tests within the community, but test results from LFT and PCR were not always uploaded to the hospital EPR, making it difficult to track results and associate these with ED and Orthoptic Department results. BMEC is a tertiary referral centre for the West Midlands therefore, some patients may have been admitted and treated for COVID-19 in hospitals closer to their homes for which there is no record on the hospital EPR at BMEC.

### Limitations

The limitations of this audit are the lack of access to information on patient’s COVID-19 status; including the initial lack of COVID-19 testing data at the start of the pandemic, and the limited access to COVID-19 test results performed in the community. This meant it was difficult to attribute the trends in presentations of ocular motility defects to confirmed COVID-19 infection. There were other confounding variables within the audit, including pre-existing conditions, increase of screen time from home working, lack of exercise, and closure of community optometry services, which may have influenced the number of presentations. This again made it difficult to attribute any trend in presentations to a particular cause. This audit was limited by having just one year available for comparison. Audits of the incidence of ocular motility defects could be improved by having multiple years of previous data to enable better analysis, greater consideration of confounding factors, and comparison of trends across multiple years.

## Conclusion

In this audit, the incidence of patients with ocular motility defects presenting to BMEC ED was higher in 2020 compared to 2019. Unfortunately, it is difficult to know whether the increase of neurogenic conditions causing ocular motility defects in 2020 is directly related to COVID-19 infection. There was a much higher incidence of trochlear nerve palsy in 2020 but we were unable to identify a direct relationship with COVID-19, as many of the patients had pre-existing conditions that increase the likelihood of cranial nerve palsy.

Further audits of the incidence of ocular motility defects during the COVID-19 pandemic will help our understanding of how COVID-19 may have affected the presentations to the ED and Orthoptic Department. Future audits should consider using multiple years of data to enable greater analysis of trends in presentation and incidence of ocular motility defects. It is also recommended that clinicians ask specifically about patient’s history of COVID-19 infection for better record keeping of infection status and ocular motility defects for future analysis.
